# In patients’ words: natural language processing of reports from patients experiencing orofacial pain and dysfunction

**DOI:** 10.1186/s10194-025-02095-z

**Published:** 2025-07-30

**Authors:** Dominik A. Ettlin, Markus Wolf, Nikola Biller-Andorno, Gerold Schneider

**Affiliations:** 1 Digital Society Initiative (DSI), University of Zurich, Zurich, Switzerland; 2 Department of Reconstructive Dentistry and Gerodontology, School of Dental Medicine, University of Bern, Bern, Switzerland; 3 Faculdade São Leopoldo Mandic, Instituto e Centro De Pesquisas São Leopoldo Mandic, Campinas, SP Brazil; 4 Department of Psychology, Clinical Psychology and Psychotherapy Research, University of Zurich, Binzmühlestr. 14, Box 16, Zurich, CH-8050 Switzerland; 5 Population Research Center, University of Zurich, Zurich, Switzerland; 6 Institute of Biomedical Ethics and History of Medicine, University of Zurich, Zurich, Switzerland; 7 Department of Computational Linguistics, University of Zurich, Zurich, Switzerland

**Keywords:** Conceptual maps, Natural language processing, Orofacial pain, Temporomandibular disorders, Text mining, Topic modeling, Web-based Interdisciplinary Symptom Evaluation

## Abstract

**Background:**

Provision of value-based, patient-centered care requires careful appraisal of patients’ symptoms. Understanding subjective experiences, needs, and feelings is crucial for shared decision-making, especially when clinical findings differ from individual perceptions. Natural language processing (NLP) offers new ways to understand patients’ perspectives. This exploratory pilot study aimed to exemplify the use of three popular NLP methods to analyze open-ended textual self-reports from individuals experiencing orofacial pain and dysfunction for a more comprehensive understanding of their subjective symptom burdens.

**Methods:**

This study used topic modeling, conceptual maps, and lexicon-based linguistic style analysis to analyze texts from 2,237 patients experiencing orofacial pain and/or dysfunction, who provided brief written descriptions of their chief complaints, functional limitations, and expectations.

**Results:**

From the aggregated text corpus of 111,923 words, unsupervised topic modeling identified 10 meaningful topics by clustering words related to prevalent complaints, constraints, and co-morbidities like tinnitus and insomnia, highlighting patients’ hopes for understanding causes and receiving a clear diagnosis. Conceptual maps of the 200 most frequent words or expressions revealed occupational limitations as significant constraints and highlighted the patients’ need for understanding causes and diagnoses. Linguistic style analyses were used to enrich the map, revealing negative emotional associations with chief complaints and the patients’ struggle to reduce uncertainty and understand their illness.

**Discussion:**

The results revealed distinct language patterns in open-ended orofacial pain reports. Chief complaints were associated with terms linked to anatomical locations and temporal patterns, functional limitations with impaired masticatory function, work-related activities and sleep disturbances, and expectations with an improved understanding of symptoms. Adding linguistic categories allowed for the validation of unsupervised methods and offered a nuanced approach to evaluate symptom burdens. NLP methods complement traditional information collection by capturing patients’ views, which are crucial for healthcare practice and shared decision-making within a biopsychosocial framework. When integrated into clinical workflows, NLP technologies might be a promising way of enhancing comprehensive symptom appraisal, benefiting both patients and clinicians alike.

**Supplementary Information:**

The online version contains supplementary material available at 10.1186/s10194-025-02095-z.

## Background

The evaluation of pain and functional limitations relies on subjective reports and objective findings. The latter are absent in some common primary or idiopathic pain types, including head, facial, and dental pain. Their phenotypic diagnostic criteria are mostly based on descriptions of the pain experience [13, 15]. Even when tissue changes are detectable, patient reports and findings may mismatch, which can result in a disconnect between patients’ concerns and their medical providers’ view. This has also been observed in the context of painful temporomandibular disorders (TMDs), which is why international experts emphasized the necessity to recognize that persistent orofacial pain arises from a complex interplay of biological, biomechanical, psychological, and social factors that defy simple explanatory models [1, 31, 34, 35, 37]. A comprehensive assessment of subjective disease burden is therefore crucial for value-based, patient-centered care.

 Limited familiarity with measures of psychological, social and pain-related burdens may result in assigning greater weight to objective findings than to self-reports. When their use is considered, low-cost questionnaires that capture self-report data in the form of check-box and Likert-scale type items are favored over more costly open-ended, narrative techniques that assess illness experiences in the form of unstructured textual data (e.g., DIPEx) [12, 32]. Yet, questionnaire sum-scores often inadequately reflect the personal context and the impairment of an individual’s well-being, both of which significantly contribute to suffering. Namely in the context of orofacial pain and dysfunction, taking the patient’s experience into account reveals valuable information that relevantly influences treatment decisions [4, 7].

Fortunately, data-driven text-based exploratory techniques are increasingly capable of transforming unstructured or weakly structured content into meaningful, quantifiable formats [20, 23, 26, 29]. Text mining, computer linguistic (CL) and natural language processing (NLP) methods rely on algorithms to automatically recognize and process human language, thus enabling tasks such as keyword extraction, topic modeling, conceptual mapping, as well as language style and sentiment analysis. By converting unstructured textual content into quantified structured data, clinical researchers can efficiently analyze large and complex datasets, thus saving time and potentially improving decision-making, although various challenges associated with unstructured data use in health research need to be kept in mind and addressed [30].

This exploratory pilot study aimed at introducing three frequently applied NLP methods for mining aggregated open-ended self-report text from individuals experiencing orofacial pain and dysfunction, and presenting resulting insights. Specifically, the aims of this study were to demonstrate their use for:

1. extracting the most common thematic clusters and assign weights to contents from a comprehensive text corpus by applying unsupervised *topic modeling*;
2. visualizing conceptual relationships in the text corpus applying weakly unsupervised *conceptual maps* for the automated analysis and visualization of single words and terms in relation to predefined anchor themes;
3. assessing potential psychological implications by applying dictionary-based *linguistic style and sentiment analysis*.

## Methods

### Data

For this retrospective observational study, we analyzed anonymized data from 2,237 patients with complaints in the oral and facial area who presented to the interdisciplinary pain unit of the Center for Dental Medicine at the University of Zurich between March 2017 and January 2020. According to Swiss law, ethical approval is not required for researching anonymized data.

Prior to their first contact with a specialist in dentistry or pain psychology, subjects completed a web-based interdisciplinary symptom evaluation (WISE; https://www.wisechoice.ai) [8]. In the WISE, unstructured data are collected from open-end texts and pain graphics, whereas validated questionnaires provide structured data [2]. For the purpose of this study, we analyzed the patients’ open-ended text entries in the following three free-text fields (anchor themes) of the WISE (Supplementary Fig. S1):

1. “Please describe your chief complaint for which you seek evaluation” (*chief complaint*). (Translated from the German original item: “*Beschreiben Sie bitte Ihre Hauptbeschwerde, weswegen Sie zur Abklärung kommen*”; Hauptbeschwerde).
2. “What functional limitations do you experience due to your chief complaints? (*functional limitations*). (German original item: “*Was können Sie wegen Ihrer Hauptbeschwerde nicht mehr tun?*”; Einschränkungen)
3. “What do you expect from the evaluations and treatments?” (*expectations*). (German original item: “*Was erwarten Sie als Resultat der Untersuchungen und Behandlungen*?”; Erwartungen)

### Natural language processing methods

For presenting the use of NLP methods for mining patients’ individual chief complaint descriptions, the following three approaches were applied: Unsupervised *topic modeling;* weakly supervised *conceptual mapping;* and dictionary-based “supervised” (i.e, rule-based) *linguistic style analysis*. Prior to the analysis, rare words and stop-words (e.g. articles and prepositions) were removed in accordance with NLP methodology. In a nutshell, an unsupervised method, topic modeling, was chosen as an efficient method to “summarize” the text into main clusters, i.e, identify thematic clusters, or topics, from large unstructured text data. While topic modeling is “by far the most popular unsupervised machine learning method in the social sciences” (p. 1464) [22], its application on open-ended patient responses has not been fully recognized. Similarly, conceptual mapping is an underused method that offers a visual representation of relationships between words, and thus, has the potential to uncover hidden conceptual relationships in large natural language data. Finally, using a dictionary-based tool like the Linguistic Inquiry and Word Count (LIWC) [17] complements data-driven analyses by adding another layer for better understanding psychological dimensions of language use.

#### Topic modeling

This unsupervised NLP method analyzes the frequency and co-occurrence of individual (key) words in a text corpus, which enables the recognition of word clusters which are called *topics* [3]*.* Topic modeling was chosen as it is a completely data-driven, unsupervised method. The clusters it forms do not depend on predefined categories but instead reveal patterns in the text data. Topics represent overarching themes, whose meaning can be inferred from their word groupings. The extraction of topics from the examined free text, is based on a Bayesian probability calculation: *p*(topic|document) * *p*(word|topic); where ‘*document’* and ‘*word’* are given, and the *topic* is iteratively fitted to the training data until an optimal probability peak is reached. Topic modeling delivers both topics and their most salient, ranked keywords.

The content of the three free text fields (anchor themes) was analyzed as an entire corpus, thus, the analysis was based on the concatenation of the three free-text fields of each individual patient. The R-package (R version 3.3.1) for structural topic model (stm, version 1.3.6) was used. Semantically meaningless stop-words (e.g. articles, prepositions) and rare words (words with a frequency < 1%) were removed prior to the analysis. To allow for additional contextual interpretation at a higher abstraction level, the topics within the anchor themes formed by the three free-text fields (chief complaints, functional limitations, treatment expectations) were juxtaposed on the basis of a logistic regression analysis which is a standard procedure of the stm package. Our choice of the number of topics, which is set manually, was based on interpretational simplicity (which gives preference to a smaller number of topics) and model diagnostics which deliver maxima and minima for established diagnostics, where held-out likelihoods and residuals are most important. According to these diagnostics, optimal values (highest held-out likelihood and lowest residuals) scored between 10 and 20 topics (Supplementary Fig. S2). For interpretational simplicity we finally decided to present a ten topics solution.

#### Conceptual maps

Conceptual maps visually represent relationships between words and concepts [9]. Their creation uses Kernel Density Estimation as an unsupervised method, or weakly supervised as in our case, if metadata information is added. One advantage of conceptual maps is that they break up the boundaries between individual topics and display the full gradience, by showing words that are shared between two topics [28]. Kernel Density Estimation calculates relative distances between words in a semantic context. The method is based on the observation that related words, e.g. *jaw, ears, tooth*, are topographically adjacent both in the physical world and in the corresponding text corpora [9]. Accordingly, the calculation of neighboring occurrence in text yields a data-driven measure of conceptual similarity with generally broad applications [27].

Kernel Density Estimates are kernel functions of these mutual co-occurrences learned from corpus data. Kernel Density Estimation is a popular smoothing method [38] that uses an approximate kernel function instead of the absolute number of co-occurrences of lexical items, resulting in smoothed counts as it smooths out data fluctuations. Conceptual maps are then calculated from the word co-occurrence matrix, which has the effect that closely related concepts form clusters in the map, while less closely related words appear increasingly distant. Words with similar content appear close together on the conceptual map. Words that occur primarily in one text field form clusters in the corners of the map. Words that occur in two text fields are recognizable as a connection between two corners, and words in all text fields are close to the center. David McClure's *textplot* tool and a Python library^1^ were used to calculate the word-word matrix of the kernel densities, and *Gephi* was used to visualize the conceptual maps.^2^ We used the spring attraction algorithm *ForceAtlas* [14] to create the map topology. In the present study, the 200 most frequently occurring words in the entire corpus were used, excluding stop-words.^3^ Since the method takes into account the structure of the entire text corpus, the three free text fields were sorted accordingly, thus adding weak supervision.

#### Linguistic style and sentiment analysis

For the linguistic style analysis as a representative of a lexicon-based or rule-based method, the Linguistic Inquiry and Word Count was applied (LIWC, German version) [17]. LIWC is one of the most frequently used standard programs for extracting linguistic and psychological dimensions from natural language. Its approach is lexicon-based and assigns individual words or word stems in a given text to more than 70 different language categories, e.g. positive or negative emotion words (*posemo, negemo*), or words relating to physical (*body*, *ingest*), cognitive (*cogproc*), and social (*affiliation*, *social*) processes. In addition, basic linguistic features such as time reference (*past*, *present*, *future*), pronouns (e.g. first person, *ipron*), punctuation marks (*interrog*) or conjunctions (*conj*) are taken into account. The language categories have been examined and validated in numerous psychological and medical studies [33]. In this study, LIWC categories were added to the conceptual map for semantic enrichment and to infer psychological implications from the patients’ open-ended text entries. Due to the exploratory and data-driven nature of this study, all LIWC categories were used in an initial step of the analysis, but in the final conceptual map only the most prevalent categories were retained that met the frequency threshold of at least 200 words (for the final list of included LIWC categories see legend of Fig. 5). Adding LIWC categories to the conceptual map means that we used a “weakly supervised” method, enriching the word-based map with known well-validated linguistic and psychological concepts, thus adding further structure to the map and providing insights into particularly relevant concepts. While areas of the map that largely stay unchanged after the addition of LIWC partly can be interpreted as a validation of the method, areas that change may point to new information, adding further data points to a triangulation perspective [21]. Complementing unsupervised methods with supervised methods is an established strategy analogous to the comparison of unsupervised principal component analysis, which delivers data-driven trends across the entire data, or to supervised linear discriminant analysis, which focuses on differences between classes.

## Results

**Fig. 1:**
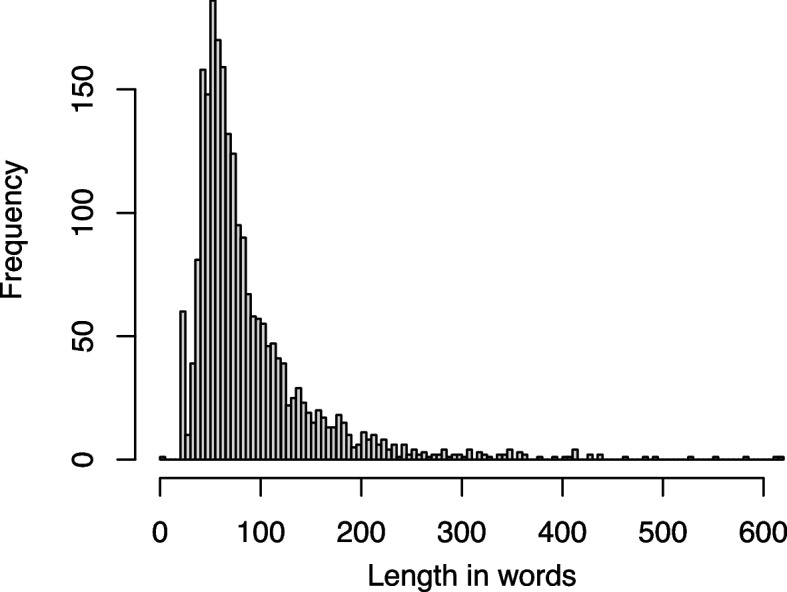
Word count distribution of text entries from the three open-ended text fields of 2,237 patients’ WISE forms

The total text corpus analyzed in this study consisted of 111,923 words (100%) written by 2,237 individual patients, with 58,159 (52%) words written in the free-text field *chief complaints*, 25,718 (23%) in the *functional limitations*, and 28,046 (25%) *expectations*. The distribution of the word lengths in the total corpus of the three open-ended free-text fields is shown in Fig. 1. Calculated for each text field of a single patient’s WISE entry, the mean word counts (Mean ± Standard Deviation) were: *chief complaints:* 31 ± 45 (median = 17), *functional limitations*: 14 ± 17 (median = 9), and *expectations*: 15 ± 18 (median = 10).

As follows, we report the results of the computational linguistic analyses, beginning with the results of the topic modeling based on the 2,237 individual patients’ WISE responses, followed by two conceptual maps that were built on the basis of the full text corpus enriched with LIWC categories as a supervised method.

### Topic modeling

**Fig. 2:**
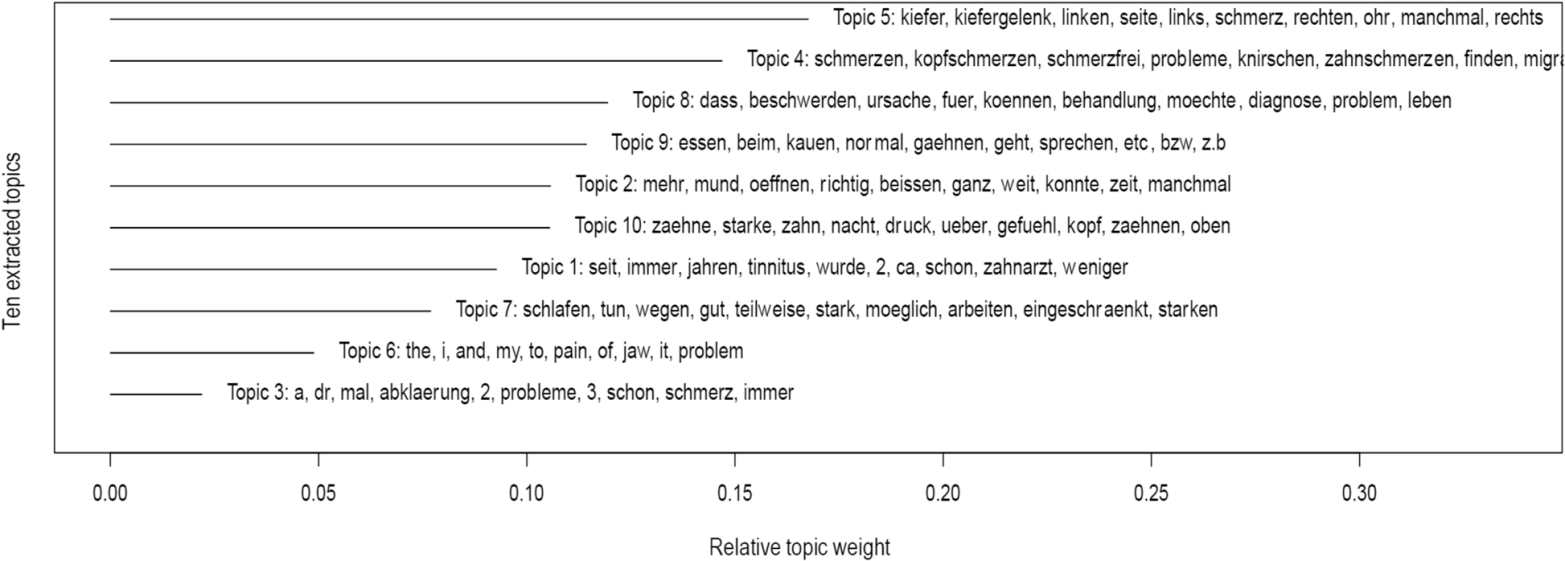
Topics extracted by unsupervised topic modeling. The y-axis represents the ten topics with topic-defining keywords. Topics were extracted from the text corpus consisting of 111,923 words. Topic count was limited to ten, whereas the algorithm arbitrarily assigned the numbers to each topic. Bars on the x-axis represent the relative weight of a given topic in the total corpus. Within each topic, keywords are listed according to their importance from left to right. English translations: Topic 5: Jaw, jaw joint, left, side, left, pain, right, ear, sometimes, right; Topic 4: Pain, headache, pain-free, problems, grinding, toothache, find, migraine; Topic 8: That, complaint, cause, for, can, treatment, want, diagnosis, problem, life; Topic 9: Eating, while, chewing, normal, yawning, possible, speaking, etc., or, e.g.; Topic 2: More, mouth, open, proper, bite, completely, because, could, time, sometimes; Topic 10: Teeth, strong, tooth, night, pressure, over, sensation, head, teeth, above; Topic 1: Since, always, years, tinnitus, has been, 2, about, already, dentist, less; Topic 7: Sleep, do, because of, good, partly, strong, possible, work, limited, strong; Topic 6: The, I, and, my, to, pain, of, jaw, it, problem; Topic 3: A, dr, times, clarification, 2, problems, 3, already, pain, always

Figure 2 shows the distribution of the extracted topics in combination with the most prevalent keywords in each topic. Note that the length of the bars represents the proportion of each topic, i.e its relative importance *p(topic)* within the complete text corpus. For better interpretation we restricted the model to a total of 10 topics. It may be confusing that their numbering is an arbitrary assignment given by the algorithm rather than an order based on their weight or importance. Also note that the algorithm allows the assignment of keywords to more than one topic (e.g., the word *pain (Schmerz*).

The fact that the most prevalent complaints presenting at the orofacial unit were masticatory myalgia, TMJ arthralgia, or a combination of these, as well as neuropathic pain, and in some, a combination of TMD and neuropathic pain [10], seems to be well reflected by the extracted topics: The most prevalent topic (*#5*), which covers approximately 17% of the total words, consists of expressions that are specifically related to the *jaw* (German: *Kiefer*), the *temporomandibular joint* (*Kiefergelenk*), or the location such as *left*, *right*, or *side.* In addition, the topic hints towards co-occuring symptoms associated with the *ear* (*Ohr*). Topic #4 reveals additional pains in various regions such as the head (*Kopfschmerzen, Migräne*) or teeth (*Zahnschmerzen*). Topic #8 on the other hand reflects expectations in terms of *treatment* (*Behandlung*), *diagnosis* (*Diagnose*), *and cause* (*Ursache*) in relation to wishes (*would like to/möchte*) or conclusions (*so that/dass*). Topic #9 summarizes content that relates to impaired masticatory system functions *(eating/essen; chewing/kauen; speaking/sprechen; yawning/gähnen*), whereas topics #2 and #10 represent anatomical locations, i.e., the *mouth (Mund; topic#2)* and quantitative aspects of its functionality (more*/mehr, fully/ganz, wide/weit, time/zeit, sometimes/manchmal*), as well as *teeth (Zähne; pressure/Druck; feeling/Gefühl;* topic #10)*. Topic#1* making up approximately 10% of the word count covers expressions that are related to the temporal development or complaint history (*since/seit; always/immer; years/Jahren; was/wurde*) which also includes chronic co-morbidities (*tinnitus/tinnitus*) and treatments (*dentist/Zahnarzt*). Topic #7 covers interferences with everyday activities (*sleep/schlafen; work/arbeiten; do/tun; restricted/eingeschränkt; strong/stark*). The final two topics are likely remnants from English speaking subjects (topic #6), and a compilation of words that clusters as a residual category (topic #3).

**Fig. 3:**
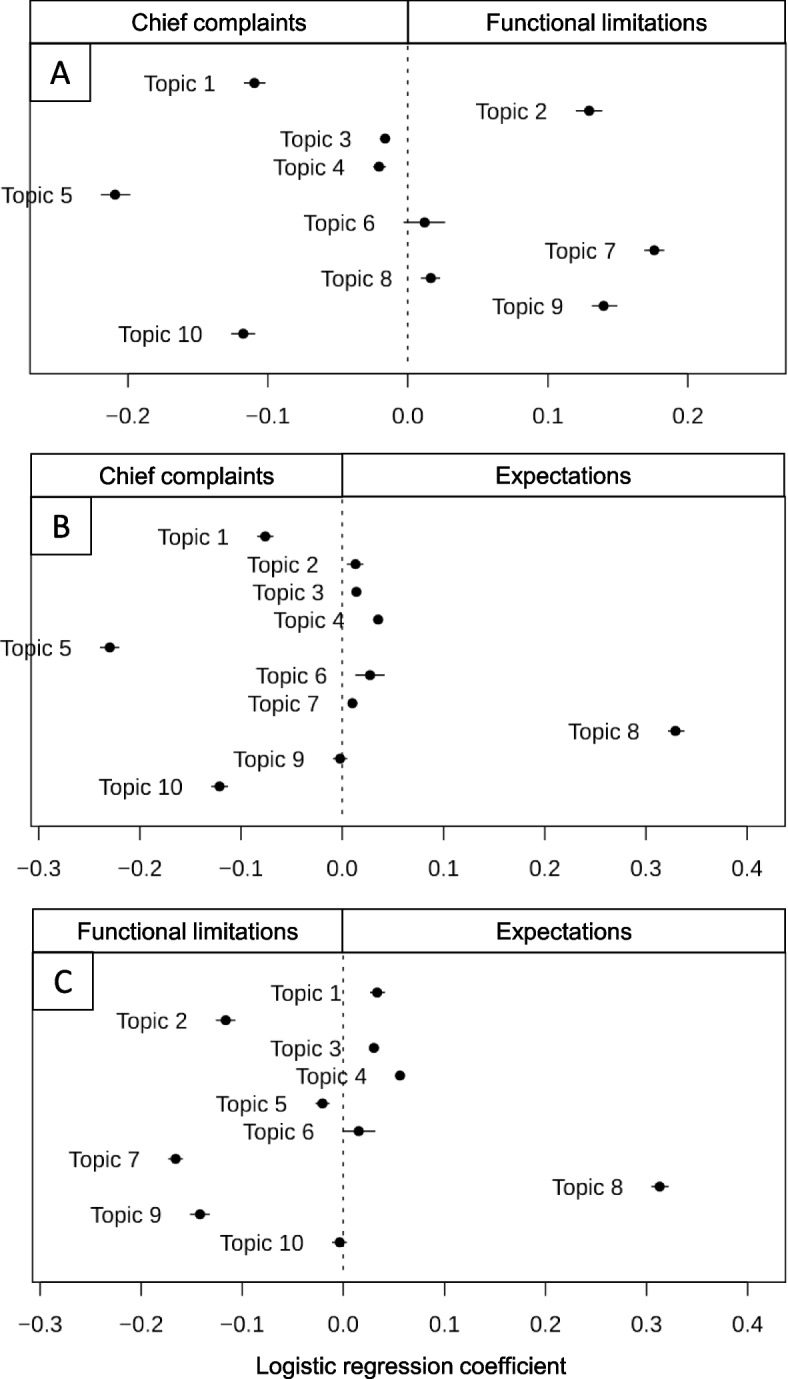
Presentation of topics within WISE free-text fields. The Figure illustrates the topics’ relative distributions among the three anchor themes. Dots and error bars represent estimated logistic regression coefficients with 95% confidence intervals (https://cran.r-project.org/web/packages/stm/index.html). The three panels show the ten topics within anchor themes according to the following juxtapositions; *A:* *Chief complaints* vs *Functional limitations; B: Chief complaints* vs *Expectations; C: Functional limitations* vs *Expectations*

In a subsequent step, we estimated the relative proportions of the 10 topics in each of the three free-text fields, using the stm library [24], which applies logistic regression to predict the binary decision between the anchor themes, e.g. *chief complaints* vs. *expectations,* and plotting the resulting feature weights (see Fig. 3A-C). When juxtaposing two anchor themes, the coefficient levels of this weakly-supervised method indicate the relative association strength of any topic among the given themes which may reveal clinically relevant patterns. First, we calculated the logistic regression coefficients for the 10 topics in the two fields *chief complaints vs functional limitations* (Fig. 3A). Topic #5 strongly leans towards the side of *chief complaints,* highlighting aspects related to location, lateralization, and temporal focus. Similarly, topic #1 referring to the illness history, and topic #10, display a stronger association with *chief complaints*. In contrast, topics #2, #7, and #9 tend to relate to *functional limitations*. The logistic regression places topics #3, #4, #6, and #8 towards the center, indicating weaker associations with either of the two anchors themes.

Figure 3B compares the 10 topics in relation to *chief complaints* vs *expectations*. Topic #8 prominently stands in proximity to *expectations,* likely indicating the patients’ hopes and expectations regarding clarification of illness causes, diagnosis and treatment options. As in Fig. 3A, topics #5 and #10 are on the side of *chief complaints*.

Finally, Fig. 3C shows how the 10 topics distribute among *functional limitations* vs *expectations*. Topics #2 and #9 addressing jaw-related limitations are clearly located towards *functional limitations*, highlighting specific activities that are impaired or not possible (anymore) such as eating, chewing, etc. Topic #7 is also located in the *functional limitations* segment, but reflects other impediments such as sleep and work. Analogous to Fig. 3B, topic #8 lies near *expectations*.

### Conceptual maps

**Fig. 4:**
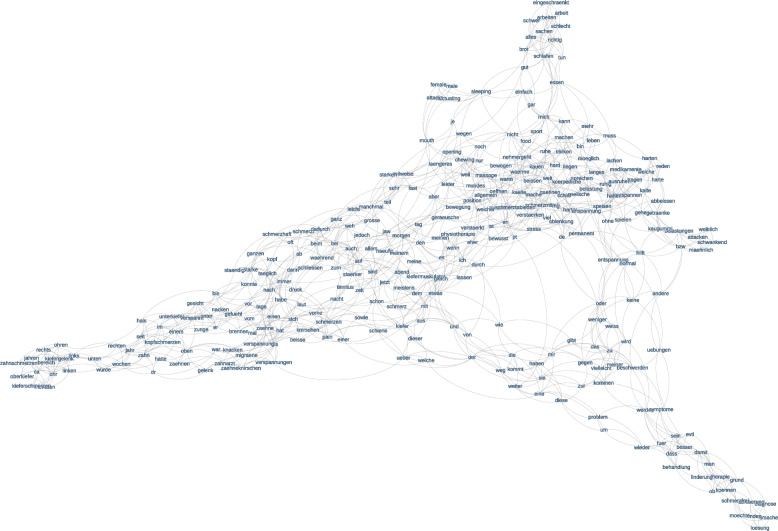
Computer-generated conceptual map emerging from the 200 most frequent words extracted from the three WISE free-text fields. For an English translation of the German terms in the Figure we refer the reader to the text

**Fig. 5:**
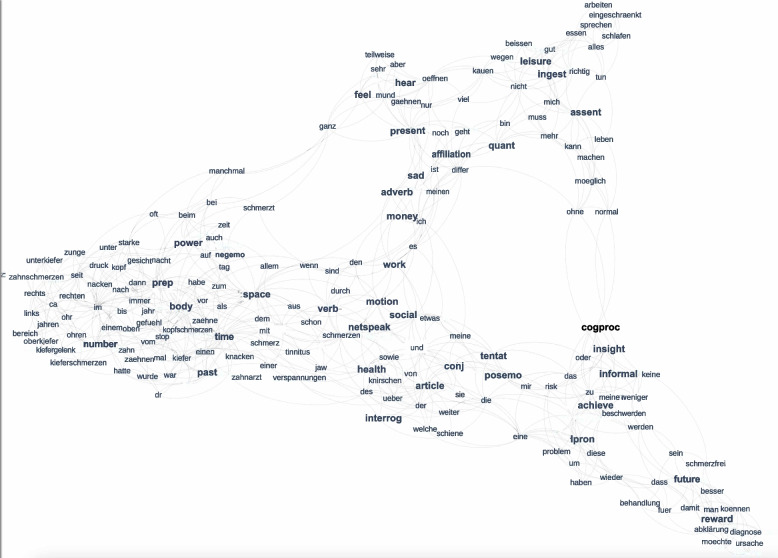
Computer-generated conceptual map emerging from 200 most frequent words or LIWC categories (boldface), respectively, extracted from the three WISE free-text fields. For an English translation of the German terms in the figure we refer the reader to the text. The following LIWC categories were retained in the map (abbreviations in brackets): Achievement (achieve); Common adverbs (adverb); Affiliation (affiliation); Articles (article); Assent (assent); Body (body); Cognitive processes (cogproc); Common verbs (verb); Conjunctions (conj); First person singular pronouns (Ipron); Future focus (future); Health (health); Hear (hear); Informal language (informal); Ingestion (ingest); Insight (insight); Interrogatives (interrog); Leisure (leisure); Money (money); Motion (motion); Negative Emotion (negemo); Netspeak (netspeak); Numbers (number); Past focus (past); Positive Emotion (posemo); Power (power); Prepositions (prep); Present focus (present); Quantifiers (quant); Reward (reward); Sadness (sad); Social processes (social); Space (space); Tentative (tentat); Time (time); Work (work). For a detailed description of the categories we refer the reader to Meier et al. [17]

In the following we present two conceptual maps that show the distribution of the 200 most frequent words of the full text corpus (Fig. 4) and with selected language categories from LIWC-based linguistic style and sentiment analysis included (Fig. 5). The translations of the German terms depicted in Figs. 4 and 5 can be found in the Supplementary Materials (Table S1). In these maps, the corners represent each of the three anchor themes (*chief complaints, functional limitations, expectations*). Words close to the corners are strongly associated with these themes, and words located in between indicate their shared appearance in more than one theme.

Figure 4 shows that words related to the anchor theme *chief complaints* are clustered on the left side of the map. In analogy to the results of the topic modeling, words in this part of the map are related to lateralization (*right (*German*: rechts), left (links)*, location (*face (Gesicht), upper jaw (Oberkiefer), lower jaw (Unterkiefer), ear (Ohr), headache (Kopfschmerzen), neck (Nacken), tooth (Zahn), tongue (Zunge)*). Also, temporal relation terms aggregate here and point towards the illness history (*was (war), year/s (Jahr/e), had (hatte), since (seit)*). In addition, words specifying various symptom characteristics such as *clicking (Knacken), tension (Verspannung), pressure (Druck), pain (Schmerz),* or *tinnitus,* can be found between this and the center part of the map. Terms in the upper right part specify the various *functional limitations* in *life (Leben)*, specifically in domains such as *working (arbeiten), speaking (sprechen), eating (essen), biting (beissen), or sleeping (schlafen).* Finally, the lower right part includes expressions such as *pain-free (schmerzfrei), can (koennen)* or *better (besser), treatment (Behandlung)* combined with a desire to understand the *cause (Ursache)* and *diagnosis (Diagnose),* reflecting the anchor theme *expectations.*

Words at the intersections that were mentioned in more than one anchor theme provide additional insights into the spectrum of potential overlap. Such bridging words between *chief complaints* and *functional limitations* refer to intensities (*total (ganz), partly (teilweise), very (sehr)*) and time dimensions (*often (oft), time (Zeit), sometimes (manchmal), morning (*Morgen*), day (*Tag), *night* (Nacht). While skewed towards *chief complaints,* the intersection to *expectations* is characterized by a semantic cluster that points towards muscular impairments (tooth *grinding (Zähneknirschen), tensions (Verspannung), pain (Schmerz), night guard (Schiene*)). At the intersection between *functional limitations* and *expectations* words are found such as *possibly (vielleicht)*, *less* (weniger), *without (ohne)*, *normal (normal)* that reflect a tentative wish for a transition from impairment to normal functioning. Finally, the low word density in the center reflects the sparsity of shared appearance of terms in all three anchor themes.

The results from the LIWC-based linguistic style and sentiment analysis are shown in Fig. 5 (for English translations see Supplementary Table S1). LIWC categories were added to re-create a map covering again the 200 most prevalent terms. While the general topology remained similar to Fig. 4, the word composition changed slightly. The linguistic concepts from LIWC appropriately fitted the word patterns; for instance, LIWC categories appearing in the *chief complaints* anchor theme (left side of the map) confirm the temporal dimension (LIWC categories *time* and *past*), physical symptoms and locations (*body*) as well as negative consequences associated with it (*negemo,* i.e., negative emotion words). The LIWC categories in the upper right of the map—*ingestion*, *leisure or affiliation—*within a mostly present-time focus *(present)* seemed to supplement functional limitations as mentioned above*.* LIWC categories located in the lower right referred to the future (*future*) with a focus on cognitive aspects reflected by *cognitive processes (cogproc)* and *insight* as well as *achieve* and a hope for positive consequences (*reward*). These categories resonated well with patients’ expectations and hopes as mentioned above.

Many words that were mentioned as both, *chief complaints* and *expectations*, referred to LIWC categories *health, social* and *motion* with positive emotions *(posemo)* skewed towards expectations. LIWC markers around insecurity, indicated by a tentative language (*tentat*), in addition to the use of interrogatives (*interrog*) and conjunctions *(conj)* likely reflect the patients’ need for information. Interestingly, the central location of *money* and *work* points towards a unifying topic.

## Discussion

The International Classification of Orofacial Pain [13] lists over 70 distinct diagnoses. The most prevalent complaints presented at the interdisciplinary orofacial pain unit, as reflected in the comprehensive data set analyzed in this study, were masticatory myalgia, TMJ pain and dysfunction, or a combination of these. Another group of patients experienced neuropathic pain, and some suffered from a combination of TMD and neuropathic pain [10]. Employing anonymized open-ended free text entries from a large cohort of patients who completed self-reports prior to their clinical presentation, this study applied state-of-the-art NLP methods to analyze these reports. This approach aimed at offering novel insights from the patients’ perspectives and visualized complex relationships between somatic and psychosocial burdens.

Among the multiple available methods, we chose to present topic modeling as an unsupervised approach and conceptual maps as a weakly supervised method while incorporating language style and sentiment analysis using LIWC as a lexicon-based method*.* For the exemplary presentation of these three text mining methods, open-ended, unstructured self-reports of 2,237 patients were merged and analyzed prior to their first clinical appointment [8]. The resulting large unstructured text corpus comprised approximately 112,000 words. By generating probabilistic associations between word patterns, topic modeling identified meaningful topics relevant to patients’ chief complaints and functional limitations (*topics #4, #5 and #9*), as well as associated burdens such as tinnitus (*topic #1*) and sleep disturbances (*topic #7)*. In the literature, the latter two comorbidities have been reported to be highly prevalent complaints in patients experiencing orofacial pain and dysfunction compared to the general population [5, 18]. Our computer-generated results thus fit with findings from clinical research that applied traditional methods in the form of standardized questionnaires. Importantly, our method further identified patients’ hopes and expectations, namely towards possible causes, a clarifying diagnosis and treatment options (topic #8). The relevance of this latter topic for patients stood out even more clearly in the direct juxtaposition of topics contained in the anchor themes *chief complaint, functional limitations,* and *expectations* (see Fig. 3A-C).

A potential clinical significance of identifying word clusters in a large clinical cohort like ours can be seen in their possibility for healthcare providers to uncover the most common or significant concerns among patients as well as the most prevalent conditions in the population that they care for. In clinical training settings, highlighting what functional limitations most commonly affect patients may help medical trainees to capture the potential impact that conditions have on their patients’ daily lives, which is critical for making informed treatment decisions. Patients' goals and the anticipated treatment outcomes may not necessarily align with clinicians' views, yet the former are important aspects of patient satisfaction and adherence. Additionally, in culturally diverse settings, the identification of language patterns may support clinicians to develop personalized and targeted communication strategies tailored to biographical backgrounds of patient subgroups. Finally, understanding and adjusting to a patient’s language on a micro-level may help in simplifying explanations and addressing specific concerns which, in turn, might improve compliance and therapeutic outcomes. From a macro-perspective, topic modelling may help uncover health trends in a given population. For instance, if certain themes emerge more frequently in specific demographic or clinical subgroups, this may point to areas that require targeted healthcare interventions or further investigation to adjust the service. In addition, word clusters may reveal psychological or emotional aspects related to patients’ complaints and treatment expectations which may help uncover emotional barriers or psychological factors affecting the illness perception.

Conceptual maps were used as a weakly supervised method for analyzing large text corpora. The method requires some preprocessing in form of selecting the number of extracted words, determining stop-words, and providing smoothing parameters. For the present text corpus, a limit was set to extract the 200 most frequent words or expressions. The conceptual maps (Fig. 4 and 5) emerged from the computer-generated assignment of these extracted words to the three text fields that served as preselected anchor themes. Empirically expected effects such as the appearance of tooth and jaw pain close to *chief complaints* appeared to validate the method. It is noteworthy, however, that besides the anticipated impaired masticatory functions, sleep disturbances and additionally work-related impairments emerged as relevant impediments. Occupational limitations associated with orofacial complaints are rarely addressed in the literature. Our study thus highlights that these patient concerns have not yet been adequately investigated and point towards new clinical research opportunities [11]. In the *expectations* corner of the map, not only a desire for symptom relief, but notably a need for understanding causes and diagnoses became again strikingly evident. Considering the inclusion of well-validated language categories from the LIWC lexicon, the markers *Tentative* (*tentat*) and *Interrogatives* (*interrog*) further underscored the struggle of patients to minimize uncertainty and understand their illness [33, 36]. The benefits of patient education and counseling for maximizing treatment compliance are well-known in daily practice [6, 19]. Extending current evidence generated by traditional research methods, both topic modeling and conceptual mapping methods provided novel scientific insights emphasizing the perspective of affected individuals.

A further strength of conceptual maps lies in their ability to visualize seamlessly interconnected semantic domains with thematic transitions and interfaces. For instance, the interpolated position of muscle tension (*Verspannung*) and pain (*Schmerz*) reflects the coupling between symptom burden and relief expectations. We further point out that many terms that appear on the conceptual map at a small distance are also closely linked to everyday semantics; expressions referring to various experiential dimensions exhibit semantic similarity, e.g., related to intensity (*much/sehr; strongly/stark*), temporality (*often/oft; sometimes/manchmal; during/während; often/häufig*), and concepts (*day/Tag; night/Nacht; evening/Abend; today/heute; now/jetzt*). The few English terms also clustered together (*female; male; sleeping; attacks; mouth; opening; chewing*), but even cross-linguistically, the kernel density estimation method recognized that words such as *hard* and German *hart,* as well as German *kauen* and *chewing,* are semantically related. Such observations demonstrate the essential functionality of this method. Traditionally, achieving the results of our study would likely have required the conduct of patient focus groups, manual transcription of medical histories, and clinicians organizing the themes. Text mining streamlines this process by classifying text according to content, intent, sentiment, and other psychologically meaningful language features. Conceptual maps can thus offer assistance for better understanding patterns of overlapping symptoms in complex cases, also within larger cohorts of patients that might serve as a reference. Future applications may allow the tracking of symptom development by mapping them over time and visualizing otherwise hidden trajectories, as well as their associations with treatment.

A general strength of linguistic analyses of natural language self-reports is their capability to reveal patients’ perspectives. Linguistic style analysis may provide clinicians with insights into patients’ emotional states or levels of distress. This may facilitate resource allocation and more targeted treatment. For example, erratic speech might reflect anxiety or confusion. Further, comparisons between cohorts experiencing pain in other body parts may offer insights into unique features of patients experiencing orofacial pain [16]; yet other linguistic comparisons may address sociocultural factors for investigating how different populations communicate their health and pain experience.

The potential benefits of sentiment analysis are worth exploring since emotional states often correlate with certain medical conditions (e.g., depression, chronic pain). These aspects may not be fully captured through traditional clinical questioning. Sentiment analysis may help detect underlying emotions in reports, such as frustration, sadness, anxiety, or hopelessness. NLP technology may therefore reveal a deterioration of persons'well-being that might otherwise be difficult to capture [2]. Thus, unobtrusive measures such as sentiment analysis may complement the assessment of patient satisfaction or dissatisfaction with current treatment and allow for personalization, particularly in cases of non-adherence or lack of treatment progress.

The findings of this study must be interpreted in light of some limitations of the methods applied. Although machine-learning methods are automated, they still require human expertise in the form of preprocessing (e.g., stop-word elimination), parameter choice (e.g., selection of topic numbers and smoothing parameters), and possibly weak supervision (e.g., provision of sorting anchors). Yet this limitation can also be seen as a strength, i.e., the ability to limit the number of automatically generated topics. The decision to use a specific number of predefined topics is subjective and often influenced by practical considerations. Alternatively, the process of establishing the optimal number of topics can be automated. Such approaches are popular but have been criticized [25]. In an attempt to balance the need for granularity (more topics) with interpretability (fewer topics), we opted for 10 topics with the effect that a “residual” topic (topic #3) remained, which was ambiguous and not clearly interpretable. A second limitation of topic modeling can be seen by comparing topics #2 and #9. As the algorithm relies on statistical patterns, these two topics were separated, although from a human coder’s perspective they seemed closely related. Hence, domain knowledge is still important for guiding the process to produce meaningful results. The algorithm-based separation of topics #2 and #9 thus highlights the complexity of computer-generated topics and the interplay between bottom-up, data-driven patterns and top-down human interpretation. The method requires informed choices to strike a balance between granularity and practicality. Finally, it must be borne in mind that differences in the length of the patients’ text entries might have affected the results.

Shifting the focus on conceptual maps, one of their strengths is the quick visual overview they can provide of the entire data. Using ChatGPT or other Large Language Models (LLM) will require careful prompting, fine-tuning, and domain specificity to accomplish the similar task in a reliable and valid answer beyond a pure text response. In addition, despite the powerful performance of LLMs, their limited context window will be challenged when processing a comprehensive text corpus with several steps to be kept in memory. Nevertheless, the complexity of the data also comes with limitations, namely the visual complexity of conceptual maps. Even though the anchoring points were limited to the three anchor themes *chief complaints*, *functional limitations* and *expectations*, their interconnectedness led to a dense and intricate map that may be challenging to interpret at a glance. To alleviate potential confusion, we set a restriction to the 200 most frequently used words for intelligibility. Yet, as a limitation, this may have resulted in an oversimplified map that missed relevant additional relationships. Finally, recognizing and emphasizing meaningful associations involves a subjective element, allowing room for diverse interpretations.

Also, the dictionary-based LIWC analysis has its own limitations. Being a rule-based method, LIWC relies on predefined, validated word categories. If an expression does not fit into these categories, it may be overlooked or misclassified. The simplification of language into predefined categories also prohibits the capture of subtle nuances, subjective meanings, or metaphoric language, therefore potentially missing deeper layers of expressions. The brevity of individual texts with a mean word count of 50 words may have exacerbated this limitation. Since LIWC lacks context awareness, it treats words in isolation without considering their surrounding context. This can lead to misinterpretations or inaccuracies in analysis, specifically in short or sparse texts. Despite these limitations, our study found LIWC to be a valuable tool for quickly and reliably analyzing clinical texts, especially in combination with unsupervised NLP methods, for a better understanding of potential psychological implications.

It must be acknowledged that both topic modeling and conceptual maps are unsupervised methods, and no gold standard exists against which evaluation and inter-annotator agreement can be measured. This could be seen as a limitation. As our study was exploratory, the topic interpretations were based on consensus among the authors based on their expertise in this field. Future studies may apply more systematic methods to quantify the levels of agreement. As a last restriction, we need to mention that variations in the length of patients' wordings may have influenced the results, potentially introducing bias in the analysis.

## Conclusions

The results of this study are novel in the sense that text mining and NLP methods conveyed patients’ views extracted from a large text corpus that was based on self-reports of an unselected clinical cohort rather than a prospective trial cohort. Computer-supported NLP provides a source of empirical-quantitative enrichment, and in our study, confirmed the relevance of a biopsychosocial patient assessment. In a next step, superimposing information from individual patients onto big data compiled from even larger cohorts may allow a better understanding of individual experiences and needs within a patient collective. Following the motto "a problem shared is a problem halved", such an approach may offer support to patients experiencing isolation during their symptom-related struggle. An even more expanded understanding of patients’ burdens can likely be obtained by combining markers extracted from natural language with scores derived from validated questionnaires and physical examinations. Comprehensive information that considers personal contexts helps synthesize knowledge about patients and therefore foster personalized care in a shared decision-making environment. Further, while LLMs cannot provide a transparent and reliable overview as shown in this study, they might be potentially useful for subtasks such as creating meaningful topic labels, or by producing an individual summary of a given patient’s location in the map. Once confidence and explainability of their results improve, there will be use cases in which LLMs will be increasingly applied in future studies.

## Supplementary Information

Supplementary Material 1.

## Data Availability

The datasets used in this study will be made available upon reasonable request.

## Footnotes

1 https://github.com/davidmcclure/textplot/

2 http://dclure.org/tutorials/textplot-refresh/

3 The limitation to the 200 most frequently occurring words was driven by the practical concern that a larger map is not feasible to be displayed for exemplification purposes.
