# Supplementary material for: In patients’ words: natural language processing of reports from patients experiencing orofacial pain and dysfunction
Source: J Headache Pain. 2025 Jul 30;26(1):172. doi: 10.1186/s10194-025-02095-z (PMC12312499; doi:10.1186/s10194-025-02095-z)
Supplement: Supplementary file 1 — Supplementary Material 1. [file 10194_2025_2095_MOESM1_ESM.docx]

Supplementary Materials

In patients’ words: Natural language processing of reports from patients experiencing orofacial pain and dysfunction


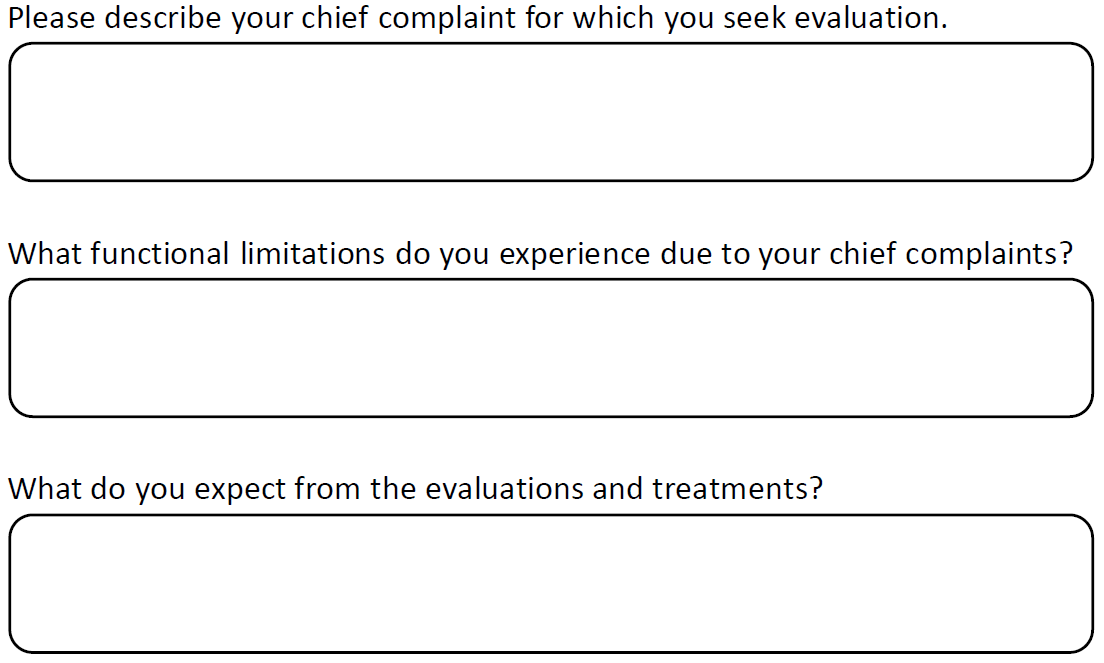


Figure S1. Three free-text fields for the open-ended self-descriptions of *patients’ chief complaints*, *functional limitations*, and *treatment expectations* in the Web-based Interdisciplinary Symptom Evaluation (WISE; Ettlin et al., 2016)


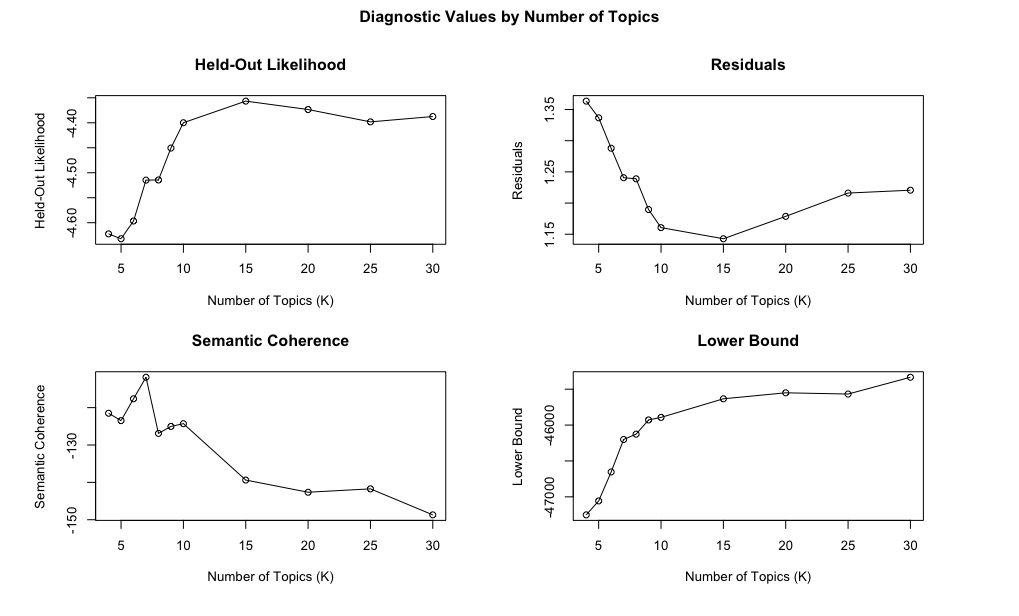


Figure S2. Diagnostic values for different numbers of topics (5≤k≤30 topics) from the stm package, according to Roberts et al. (2019)

Table S1. English translations of the German terms appearing in Figures 4 and 5 (in order of their appearance starting from the lower left of the conceptual map).

| German Original Term | English translation |
| --- | --- |
| ohren | ears |
| rechts | right |
| rechten | right |
| jahr | year |
| kopfschmerzen | headache |
| das | the |
| links | left |
| jahren | years |
| kiefergelenk | jaw joint |
| unten | below |
| wurde | been |
| hatte | had |
| dr | doctor |
| ueber | above |
| welche | which |
| zahnschmerzen | toothache |
| bereich | area |
| wochen | weeks |
| zahnarzt | dentist |
| gegen | against |
| vielleicht | perhaps |
| ca | circa |
| ohr | ear |
| kieferschmerzen | jaw pain |
| linken | left |
| gelenk | joint |
| meiner | my/mine |
| kommen | come |
| oberkiefer | upper jaw |
| hals | neck |
| seit | since |
| sich | itself |
| beisse | bite |
| verspannungen | tension |
| zaehneknirschen | teeth grinding |
| und | and |
| zahn | tooth |
| oben | up |
| zaehnen | teeth |
| mir | me/myself |
| zu | to |
| uebungen | exercises |
| beschwerden | complaints/pain |
| werden | be |
| symptome | symptoms |
| gesicht | face |
| unterkiefer | lower jaw |
| verspannt | tense |
| unter | below/under |
| gefuehl | sense/sentiment |
| nacht | night |
| oder | or |
| weniger | less |
| weiss | know |
| keine | no |
| wird | will |
| bis | until |
| nach | after |
| habe | have |
| vor | before |
| tage | days |
| druck | pressure |
| laut | loud |
| meistens | mostly |
| schmerz | pain |
| andere | others |
| nacken | neck |
| vorne | front |
| schmerzen | pain |
| staendig | constantly |
| starke | strong |
| konnte | could |
| taeglich | daily |
| immer | always |
| sind | are |
| jetzt | now |
| schon | already |
| abend | evening |
| kiefermuskulatur | jaw muscles |
| einen | one |
| einem | one |
| zunge | tongue |
| er | he |
| brennen | burn |
| mal | times |
| zaehne | teeth |
| hat | has |
| knirschen | grinding |
| sowie | as well as |
| vom | of |
| verspannung | tension |
| als | as |
| war | was |
| knacken | crack |
| migraene | migraine |
| ganzen | whole |
| kopf | head |
| meinem | my |
| meine | my |
| hilft | helps |
| dadurch | thus |
| schmerzhaft | painful |
| schmerzt | hurts |
| ist | is |
| stress | stress |
| haben | have |
| sie | you/it |
| eine | one |
| kaugummi | chewing gum |
| belastungen | burdens |
| weiblich | female |
| oft | often |
| ab | after |
| dann | then |
| schliessen | close |
| den | the |
| eher | rather |
| ich | I |
| lassen | let |
| bei | at |
| waehrend | during |
| beim | at |
| auch | also |
| allem | all |
| haeufig | often |
| einer | one |
| gibt | gives/there is |
| ganz | completely |
| auf | on |
| staerker | stronger |
| tinnitus | tinnitus |
| zeit | time |
| verstaerken | increase |
| viel | much |
| spielen | play |
| ohne | without |
| zum | to |
| es | it |
| gleich | presently |
| dem | the |
| etwas | something |
| mit | with |
| aus | from |
| schiene | splint |
| kiefer | jaw |
| dieser | this |
| durch | by/through |
| von | from/by |
| der | the |
| entspannung | relaxation |
| normal | normal |
| weh | sore |
| tag | day |
| morgen | morning/tomorrow |
| bewusst | aware |
| wie | as |
| die | the |
| weg | away |
| kommt | comes |
| weiter | furhter |
| sehr | very |
| teil | part(ly) |
| manchmal | sometimes |
| fast | almost |
| mundes | mouth |
| oeffnen | open |
| allgemein | generally |
| kaelte | cold |
| gaehnen | yawn |
| belastung | burden |
| mache | make |
| schlaf | sleep |
| seelische | psychologically |
| kalte | cold |
| halt | indeed |
| entspannen | relax |
| leicht | lightly |
| grosse | big/great |
| jedoch | although |
| bewegung | movement |
| geraeusche | noise |
| verstaerkt | increased |
| physiotherapie | physiotherapy |
| schmerzmittel/schmerztabletten | pain killer(s) |
| speisen | food |
| anspannung | tension |
| starken | strong |
| teilweise | partly |
| bewegen | move |
| weil | because |
| massage | massage |
| waerme | warmth |
| langes | long |
| weit | far |
| sprechen | speak |
| medikamente | medication |
| weiche | soft |
| gut | good/well |
| einfach | simple/simply |
| gar | even |
| essen | eat |
| nicht | not |
| nehmen | take |
| geht | goes |
| muss | must |
| laengeres | longer |
| leider | unfortunately |
| noch | still |
| nur | only |
| kauen | chew |
| liegen | lie |
| lachen | laugh |
| harten | hard |
| reden | talk |
| aber | but |
| position | position |
| abbeissen | bite off |
| gehen | walk |
| getraenke | beverage |
| meinen | my/mine |
| wenn | if |
| permanent | permanent(ly) |
| attacken | attacks |
| schwankend | fluctuating |
| bzw | respectively |
| maennlich | male |
| je | each |
| wegen | because |
| mich | me/my |
| kann | can |
| machen | make |
| ruhe | calm/still |
| trinken | drink |
| mehr | more |
| leben | live |
| bin | am |
| moeglich | possible |
| weicher | soft |
| harter | hard |
| ablenkung | distraction |
| warm | warm |
| beissen | bite |
| tragen | carry |
| harte | hard |
| koerperliche | physical |
| ruhig | calm/still |
| ausruhen | calm down |
| sport | sport |
| eingeschraenkt | restricted |
| arbeit/arbeiten | work |
| schwer | heavy |
| schlecht | bad |
| sachen | things |
| alles | all |
| brot | bread |
| schlafen | sleep |
| tun | do |
| richtig | correct |
| diese | this |
| zur | to |
| problem | problem |
| um | to |
| sein | be |
| evtl | eventually |
| fuer | for |
| dass | that |
| wieder | again |
| besser | better |
| damit | so that |
| man | one |
| behandlung | treatment |
| linderungtherapie | pain treatment |
| grund | reason |
| ob | whether/if |
| koennen | can |
| schmerfrei | pain free |
| abklaerung | evaluation |
| diagnose | diagnose |
| moechte | would like |
| finden | find |
| ursache | cause |
| loesung | solution |

References

Ettlin DA, Sommer I, Brönnimann B, et al. Design, construction, and technical implementation of a web-based interdisciplinary symptom evaluation (WISE) - a heuristic proposal for orofacial pain and temporomandibular disorders. J Headache Pain. 2016;17(1):77. doi:10.1186/s10194-016-0670-5

Roberts ME, Stewart BM, Tingley D. stm: An R Package for Structural Topic Models. J Stat Softw. 2019;91(2):1–40. https://doi.org/10.18637/jss.v091.i02
